# FedMed: A Federated Learning Framework for Language Modeling

**DOI:** 10.3390/s20144048

**Published:** 2020-07-21

**Authors:** Xing Wu, Zhaowang Liang, Jianjia Wang

**Affiliations:** 1 School of Computer Engineering and Science, Shanghai University, Shanghai 200444, China; liang_zwang@shu.edu.cn (Z.L.); jianjiawang@shu.edu.cn (J.W.); 2 Shanghai Institute for Advanced Communication and Data Science, Shanghai University, Shanghai 200444, China

**Keywords:** federated learning, language modeling, communication efficiency, topK ranking

## Abstract

Federated learning (FL) is a privacy-preserving technique for training a vast amount of decentralized data and making inferences on mobile devices. As a typical language modeling problem, mobile keyboard prediction aims at suggesting a probable next word or phrase and facilitating the human-machine interaction in a virtual keyboard of the smartphone or laptop. Mobile keyboard prediction with FL hopes to satisfy the growing demand that high-level data privacy be preserved in artificial intelligence applications even with the distributed models training. However, there are two major problems in the federated optimization for the prediction: (1) aggregating model parameters on the server-side and (2) reducing communication costs caused by model weights collection. To address the above issues, traditional FL methods simply use averaging aggregation or ignore communication costs. We propose a novel Federated Mediation (FedMed) framework with the adaptive aggregation, mediation incentive scheme, and topK strategy to address the model aggregation and communication costs. The performance is evaluated in terms of perplexity and communication rounds. Experiments are conducted on three datasets (i.e., Penn Treebank, WikiText-2, and Yelp) and the results demonstrate that our FedMed framework achieves robust performance and outperforms baseline approaches.

## 1. Introduction

With the emerging breakthroughs of new industrial revolution—Industry 4.0 and Internet of Things (IoT) technologies, society is stepping into a smart era where all objects are enclosed with the network of interconnectivity and automation by the intelligent digital technique. Meanwhile, there is a surge of heterogeneous data in edging devices: from real-time sensor operation logs to users’ data. However, data is subject to attack and poisoning easily during transferring process. This makes machine learning more challenging. Through machine learning techniques, wearable devices such as the smart band, mobile devices can be used for health status management [1,2], smart querying [3] and preference recommendation [4], etc.

In general, the virtual keyboard can recommend several probable next words options while the user is inputting on a mobile device such as the smartphone, iPad, or laptop. Language models—in particular, those using recurrent neural network (RNN) [5]—demonstrated exceptional performance in word prediction tasks [6,7,8]. Of RNN, Long Short-Term Memory (LSTM) [9] has a fine generalization in recognizing semantics with a variably sized sliding words window. Conventional language model learning is a kind of centralized approach in which all scattered devices data is sent to the server for training. However, the data from tens of thousands of mobile devices is so incredibly enormous that communication costs are extraordinarily expensive and the server can hardly meet the enormous demand for storage. Additionally, users’ data is highly private and sensitive but faces the risk of data fraud and leakage while transferring between the central server and edge-devices.

**Figure 1:**
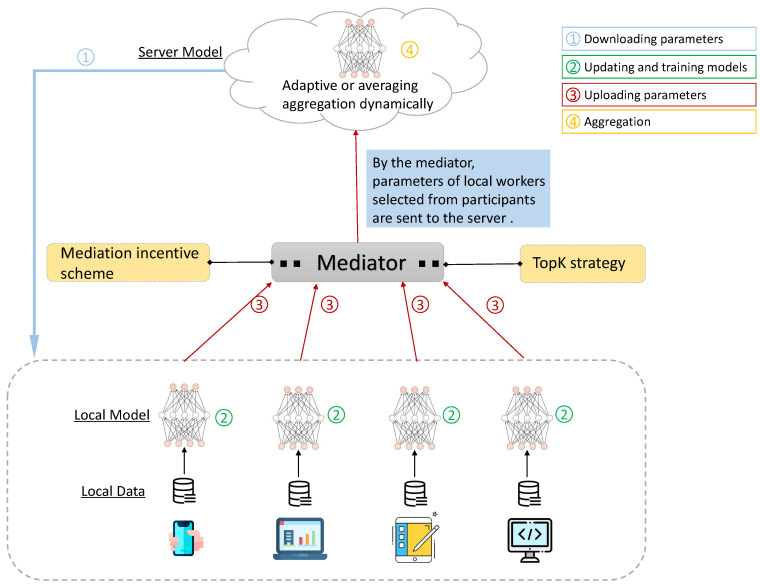
The illustration of how FedMed methodology works. Stage 1 indicates local workers download global model to devices; stage 2 represents worker models training on local data; stage 3 shows local workers upload neural network model parameters to the Mediator; and stage 4 stands for the global server aggregating worker model parameters.

Recent years have witnessed the advent of federated learning technique alongside the challenges and threats [10,11]. Through training language models cooperatively on mobile devices instead of uploading data to the server and learning from distributed models, FL lessens the high data transferring cost and privacy risk [12]. Generally speaking, FL is mainly composed of three phases. In the first phase, the global server initializes model parameters and then device workers download the model. Secondly, each worker trains the model on its local data independently. In the third phase, all local trained models are uploaded to the server via a secure protocol tunnel and aggregated for a global model. The process consisting of three phases can be iterated a great number of rounds until the global model converges or the threshold level is achieved. Even with this efficient training pattern, a crucial issue is the gigantic communication overhead generated from the model parameters exchange [13,14]. As of writing, federated learning has three researching domains: optimization design, communication efficiency, and security algorithm. To deal with aggregation and communication cost issues, we propose a novel framework, namely Federated Mediation (FedMed), as is illustrated in Figure 1.

In stage one, the neural network model on the server-side is initialized with the presetting and dispensed to local workers online in the federated network. Please note that those workers who do not get authorized by the server are invalid and excluded from the federated network. After downloading the server model, each worker trains the model to converge on local data at stage two. At stage three, parameters are uploaded to the mediator which comprises two parts, i.e., mediation incentive scheme and topK strategy. More details of the mediator can be found in Section 3. Then at stage four, the server aggregates models from the mediator to generate one new server model. Our framework design is motivated by the present bottleneck and imperfection in federated learning. In the FedMed, adaptive aggregation is introduced to make scores for each local worker model rather than averaging model weights, and topK strategy is designed to select the best local models. Then the mediator dynamically determines which kind of aggregation method and strategy is adopted. Finally, the server model aggregates local parameters that are designated by the method mediator. The primary contributions in our work are as follows:

- We design a novel federated learning framework, namely FedMed, to improve the overall performance in language modeling.
- We first propose the adaptive aggregation and mediation incentive scheme for efficient aggregation and the topK strategy to reduce communication cost, respectively.
- A range of experiments are conducted on the FedMed. Its effectiveness and generalization is proved on three benchmark datasets.

The structure of this work is arranged in the following. Section 2 introduces related works and up-to-date scholar leading edge upon federated learning. In Section 3, the FedMed methodology is presented, including adaptive aggregation, mediation incentive scheme, and topK strategy. Section 4 describes experimental settings and results on the methodology. In the end, Section 5 presents our conclusions about the proposed FedMed framework.

## 2. Related work

### 2.1. Federated and Distributed Learning

Federated learning was first termed and brought to light by Google in 2016 [15,16,17] as a decentralized machine learning theory that allows distributed training on a large scale of data in edge-devices such as smartphones, sensors, and electronic meters. Bonawitz et al. [18] introduced high-level system design at scale on production conditions and analyze present challenges and problems. Yang et al.  [19] depicted the necessary abstraction, architecture, and various applications of federated learning from an overview perspective. FL technique earned a remarkable reputation in many pragmatic fields such as visual object detection [20], health records [21], big data analysis [22], control policies [23], and medical text extraction [24].

In model aggregation, McMahan et al. [17] proposed a federated averaging (FedAvg) algorithm, which has gained enormous attention and been adopted in practical applications. However, it only computes the average worker model parameters as a global model parameters each round. Peng et al.  [25] calculated mutual information of two worker models and suggest the averaging method is not the optimal way based on entropy theory. Yurochkin et al. [26] used a Bayesian nonparametric approach that allows the model to generate expressive matching weights for training Multilayer Perceptron network (MLP) on image datasets. Ji et al. [27] introduced an attentive mechanism (FedAtt) for layer-wise model aggregation to shrink the weighted distance between the server and worker models. Additionally, communication efficiency is a key issue of federated learning at present. Yao et al. [28] presented a two-stream model rather than a single model for training to alleviate resource constraints with the maximum mean discrepancy principle each iteration. Vogels et al. [29] introduced a novel low-rank gradient compression for power layer iteration to aggregate models rapidly and perform wall-clock speedups. Lin et al. [30] found that more than 90% of the gradient information in federated SGD is superfluous and apply momentum correction, factor masking, and local clipping to the gradient compression.

### 2.2. Language Modeling

The LSTM relative techniques achieved exceptional performance in language modeling. Gerz et al.  [31] consider edsubword contexts and present a fine-tuning LSTM model to promote word prediction. Lam et al. [32] introduced the Gaussian process into LSTM language models to learn parameters uncertainty and optimum neural network gates. Ma et al. [33] use a series of univariate LSTMs to train on asynchronous temporal sequences with multivariate input for prediction. Aina et al.  [34] studied English words’ lexical ambiguity and investigated the hidden representations of contextual information in LSTM layers, which need further improvements.

## 3. Proposed Method

In this section, we first introduce preliminaries of federated learning on coexisting aggregation methods and subsequently propose the FedMed framework(i.e., adaptive aggregation, mediation incentive scheme, and topK strategy) theoretically for federated optimization in text modeling. Additionally, differential privacy technique [35] is merged into federated learning to enhance model security and avoid data leakage.

### 3.1. Preliminaries

Federated learning discloses the trade-off of model training and data storage to tackle the dilemma of training and data distribution in the IoT and distributed system domains. Basically, the server-side aggregation and the edge-side training are two critical parts of federated learning system. The device workers download the neural network model initialized by the global server to local data storage, and embark upon training under server parameters for several epochs independently. Then, the global server aggregates decentralized models uploaded from device workers for federated optimization, which holds a significant role in the neural network learning. The process of parameters uploading and downloading can be regarded as one round of communication. Thus, it can be seen that aggregation optimization is growing an attractive part in federated learning. Known as a well-established algorithm for model aggregation, FedAvg is proposed and heuristic from the baseline algorithm *FederatedSGD (FedSGD)*. In theory of FedAvg, it is hypothesized that each piece of distributed data keeps a same weighted contribution, so that the worker parameters can be averaged to build the global server. During FedAvg learning, per worker calculates gradients in one iteration. Worker *u* has local data examples $n_{u}$, and the global data sample size is *n*. Then, the central server aggregates worker weights and implements the following update:

(1) $$w=\Sigma \frac{n_{u}}{n}w^{u}$$

Stochastic gradient descent (SGD) is used to calculate the objective loss and converges on the minima upon each round of local worker training. After a set of local epochs, each selected worker weights are uploaded to the parameter server via secure computation protocols transferring for further  aggregation.

In the thorough federated learning process with averaging aggregation, the authority server launches a modern training task initialized with a presetting of model parameter weights *w*. Each of the u=1,2,…,U workers selected by server downloads the primary model with weights and hyperparameters from parameter server. Then each of the u=1,2,…,U workers launches local model training, computes gradient $g_{u}=\nabla L\left(w\right)$ and updates weights $w_{t+1}^{u}$ based on local worker data in every iteration. Finally, the parameter server computes global model weights $w_{t+1}$ by averaging all local parameter weights $w_{t+1}^{u}$ collected from distributed worker devices to optimize the global model per round of communication. This process is iterated many times until a threshold setting (e.g., loss stopping or iterations) is triggered. In brief, we present the federated learning details on distributed device workers and the common aggregation algorithm FedAvg as preliminaries of our method.

### 3.2. Adaptive Aggregation in FedMed

Federated optimization holds a significant role upon the server side in federated learning to aggregate model weights. In this paper, a novel federated aggregation method is proposed to learn model parameters from decentralized worker devices, which is regarded as adaptive aggregation. For federated learning training, each layer of models may contribute dissimilar ratio in authorized workers, and thus adaptive ratio estimation is introduced for aggregating worker models to build the server model.

The motivation of federated optimization is to find an optimum way to aggregate parameters of worker devices. Recent mass of pragmatic federated learning applications have adopted FedAvg for models aggregation; in fact, many improvements can be amenable to aggregation of models by vanilla federated averaging. Thus, we raise the optimization of federated aggregation by starting from FedAvg naturally. Each worker model is rendered identical impact on global model by FedAvg despite different worker devices. Theoretically, local training models should be given distinctive ratios due to different data distribution.

In language modeling, the context in one sentence should be considered for word prediction. In addition, the feature space composed of word vectors differs in heterogeneous data. As a matter of course, the similarity between feature spaces is not always consistent, and thus the global feature space should be constructed in a personalized pattern. To march on our Adaptive Aggregation, we study and measure the disparity between server and worker models. The process of computation relies on two key parameters: $w^{l}$, the weights of *l*-th layer on the server-side model; and $w_{k}^{l}$, the weights of *l*-th layer of *k*-th worker on the edge-side. The variant of KL divergence, i.e., Jensen-Shannon divergence is used for dealing with the asymmetric problem of KL divergence, and each round of disparity between two matrices is given by:

(2) $$d_{k}^{l}=JS\left(w^{l}\|w_{k}^{l}\right)=\frac{KL(w^{l}\|\left(w^{l}+w_{k}^{l}\right)/2)}{2}+\frac{KL(w_{k}^{l}\|\left(w^{l}+w_{k}^{l}\right)/2)}{2}$$

Then, the ratio of each worker model is recorded by calculating the disparity with the softmax function to implement the aggregation. Please note that the ratio of worker model is layer-wise, and so each round applies to the adaptive computation. The projection of probability space can be written as:

(3) $$\gamma_{k}^{l}=softmax\left(d_{k}^{l}\right)=\frac{e^{d_{k}^{l}}}{\Sigma_{k=1}e^{d_{k}^{l}}}$$

According to the Equation (2) and (3), it can be found that the model ratio $\gamma_{k}^{l}$ has a positive relation with the matrices disparity $d_{k}^{l}$, which represents the worker model holds a more important weight for global aggregation when it shows higher difference from the server model. For each worker model, we get a non-parameter ratio array $\gamma_{k}=\left\{\gamma_{k}^{0},\ldots ,\gamma_{k}^{l}\right\}$. With the ratio array of models, server can build global model based on the SGD optimization. In one round of deep learning optimization, the weights of SGD can be updated as:

(4) $$w_{t+1}\leftarrow w_{t}-\eta \nabla L\left(w_{t}\right)$$

As a remarkable optimizer, the SGD algorithm is widely used to aim at reaching a local minimum in the context of machine learning of non-linear neural networks such as RNN and CNN. Considering the SGD theoretical universality and rapid convergence downhill, we apply this to the server model for parameters optimization. The weights of server model update each round can be defined as:

(5) $$w_{t+1}\leftarrow w_{t}-\eta \Sigma_{k=1}^{u}\gamma_{k}^{l}\nabla L\left(w_{t}\right)$$

where η is the learning rate and $w_{t}$ is the global server parameters at the time of round *t*. Hence, the formula of adaptive aggregation in FedMed is given to learn the global server model. Instead of the vanilla average method, each weighted score of worker model is calculated dynamically during the aggregation process. The server implements the estimated gradient downhill on the weighted parameters by SGD. From this point of view, gradient updating is computed in a distributed way and adaptive to different workers. The novelty of our proposed adaptive aggregation in FedMed is as follows: (1) Given the different distance between the server model and worker model, the contribution to global model is evaluated in the feature space. The server can learn the word representations effectively in the layer-wise way. (2) Through the distributed gradient updating, the global model can arrive at the minimum downhill in a reasonable way and reduce the differences with worker models  comprehensively.

### 3.3. Mediation Incentive Scheme in FedMed

For building a robust aggregation, we explore the mediation incentive scheme based on our adaptive aggregation and FedAvg aggregation. When worker models are initialized by server parameters, each of them has significant disparity with regard to model weights because of scattered device data distribution. However, with rounds of model training, the gradient of loss L(·) over device data gradually converges to a smooth phase. Thus, the training process renders the server model more generalized and robust, with the disparity miniature and diminutive among worker models. The adaptive aggregation we proposed can have a better representations learning in theory. Although the FedAvg algorithm is not the optimum federated aggregation method, it has been applied to a great many of machine learning applications and performs well on identical distribution data. We are inspired by the Dropout technique [36], which introduces a simple but worthwhile randomization method for neural networks. The randomization can prevent the model from a solidifying structure that may lead to an overfitting network easily, and keep knowledge representations flow smoothly.

**Figure 2:**
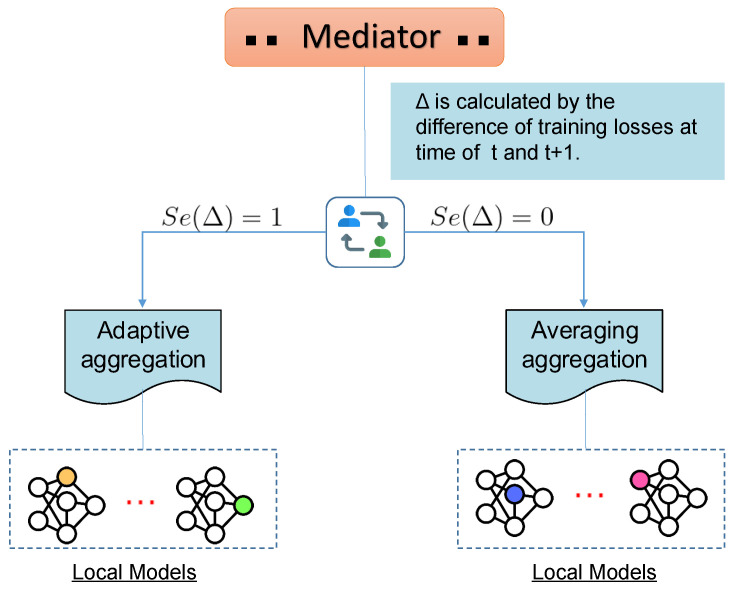
The illustration of how the mediation incentive scheme works.

Hence, we consider the incentive combination of FedAvg and adaptive aggregation to improve the training process. Through the alternative of two algorithms dynamically, randomization is introduced to avoid one specific method that can deteriorate the aggregation performance and global model’s generalization. As a matter of course, the server can learn the embedding feature space preferably with the alternate aggregation method. As is shown in Figure 2, after each round of local training, the server model calculates the training loss to compute the difference compared to last one. The training loss of server model is achieved by averaging local model losses. The incentive scheme is designed based on alteration between two adjacent loss values. The scheme on how to proceed the alteration can be defined as:

(6) $$\begin{array}{c} \Delta \leftarrow |L\left(w_{t+1}\right)-L\left(w_{t}\right)| \end{array}$$

(7) $$\begin{array}{c} Se\left(x\right)≜\left\{\begin{array}{cc} 1, & \mathrm{if}\;x\geq \epsilon ;\\ 0, & \mathrm{if}\;x<\epsilon . \end{array}\right. \end{array}$$

In the equation above, where $L(w_{t})$ means the loss of training at the round of *t*; Δ is the difference value between $L(w_{t+1})$ and $L(w_{t})$; and ϵ denotes the threshold value to discriminate which aggregation method model can choose. The Δ is seen as the independent variable *x* of function Se(·) to be compared with the ϵ. Se(Δ) equals 1 when Δ is greater than or equals to ϵ. Note the ϵ, rather than an invariable parameter, can be manipulated by different models and data sets. The procedure can be regarded as a discriminator to implement the scheme. It can give judgement on selecting either FedAvg or adaptive aggregation we proposed each round. The trained server model is more generalized than local workers in parameter space by moderating two aggregation algorithms, and can learn from federated local workers better. The complete pseudo-code of our proposed optimization method is given in Algorithm 1, of which the critical part can be found from Line 10 to Line 16.

**Algorithm 1** FedMed. *p* is the size of workers for training, *E* is the size of local epochs; the *U* workers are indexed by *u*, and S indicates the choice of adaptive aggregation or FedAvg.1: **procedure** SERVERUPDATE: 2:    Initialize $w_{0}$ 3:    **for** each round k=1,2,…
**do** 4:        p← max (1,C·K) 5:        $U_{k}\leftarrow$ (random *p* workers) 6:        **for** each worker $u\in U_{k}$
**in parallel do** 7:           $w_{t+1}^{u}\leftarrow$ WorkerUpdate $\left(u,w_{t}\right)$ 8:        **end for** 9:        //Implement mediation incentive scheme 10:        Δ← alteration of two losses 11:        S←Se(Δ) 12:        **if**
S is 1 **then** 13:           $w_{t+1}\leftarrow$Adaptive aggregation 14:         **else** 15:           $w_{t+1}\leftarrow \Sigma_{u=1}^{p}\frac{n_{u}}{n}w_{t+1}^{u}$ 16:        **end if** 17:    **end for** 18: **end procedure**

19: **procedure**
WorkerUpdate(u,w) 20:    //Run on worker *u* 21:    B← (split data $P_{u}$ into batches size of *B*) 22:    **for** each local epoch *i* from 1 to E
**do** 23:        **for** batch b∈B
**do** 24:           w←w−η∇L(w) 25:        **end for** 26:    **end for** 27:     return *w* to server 28: **end procedure**


In short, our proposed mediation incentive scheme in FedMed is introduced firstly to prevent aggregation degrading and boost generalization via the alternative mechanism based on the adaptive aggregation and FedAvg algorithms.

### 3.4. TopK Strategy in FedMed

In this subsection, another improvement—topK strategy of FedMed —is presented to alleviate the high communication cost of federated learning. Model parameters transferred between server and device workers exhaust most of the communication bandwidth. Tremendous model parameters have undermined communication efficiency highly and rendered poor performance of federated learning. To exploit finite network bandwidth and reduce the model converging time, federated learning should be communication efficient.

**Figure 3:**
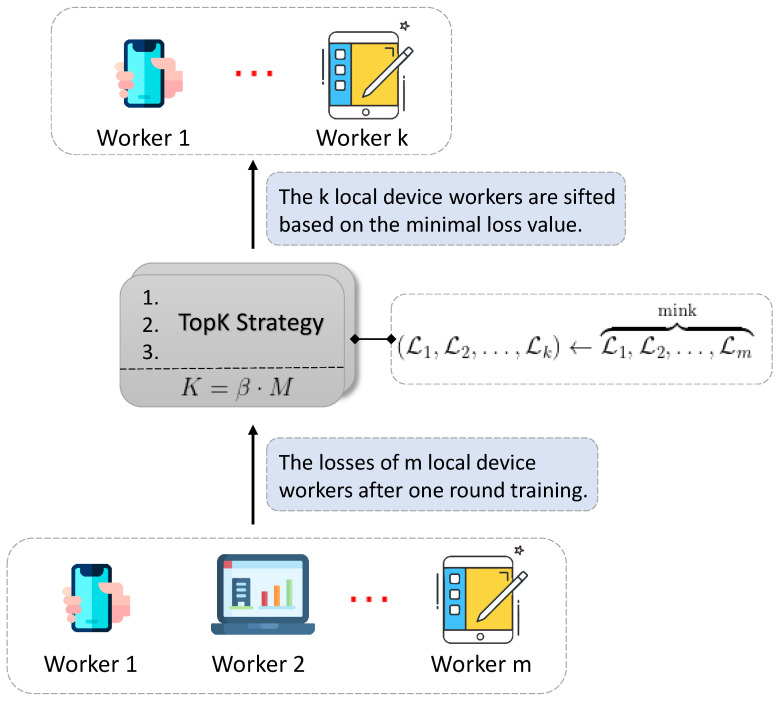
The illustration of how TopK rankings strategy works.

To trade a higher efficiency in FL, we are motivated by the topK strategy applied widely to the recommender system, which suggests several most probable and suitable items, products, or advertisements to users by the specific evaluation metric. Edge devices in federated learning are non-identical due to different local data, and some of which can be low-quality and inferior. It is reasonable to abort those workers who have poor performance during model aggregation. Hence, the topK reputation strategy of testing cost loss is adopted for federated optimization coordinating with FedMed. We incorporate topK approach into FedMed framework and make a little variation. We consider the worker to be reputable who obtains lower loss while training, and collect it into the topK category. In terms of training, the topK in the profile serves as a selector to choose the top K most preferable device workers as is illustrated in Figure 3.

Each round local workers in the set W={1,2,…,m} take part in model training, and the loss on the dataset of each worker is computed at the tail of local training. All models’ loss values in the form of an array are uploaded to the mediator of FedMed. Afterwards, the topK strategy in the mediator is applied to the training workers based on loss values and an ordered permutation is generated. Then, each of the top K workers who have lower losses is authorized to update model parameters to the global server for aggregation. The set of K workers is denoted as $\tilde{W}=\left\{1,2,\ldots ,k\right\}$. Instead, the rest of workers whose model parameters are not transferred to the server make no contribution to server aggregation, so it can highly alleviate the communication bandwidth burden and boost training efficiency. In short, the variation in topK strategy can be expressed as:

(8) K=β·M

(9) $$P=minK\{L_{1},L_{2},\ldots ,L_{m}\}$$

In Equation (8), the process of computation is subject to two key parameters: β, one hyperparameter to represent the fraction of workers to be selected; and M, the number of training workers upon each round. Note the β is in the range [0.0…1.0], where bound 0.0 and 1.0 represent, respectively, totally negligible and whole workers. We also take K as one hyperparameter in the FedMed. In Equation (9), the right part indicates that the K minimum losses are sifted from the total losses set, and the correlative workers are indexed and authorized for subsequent model transferring. In conclusion, we introduce the topK strategy into the federated learning for the first time, towards the objective of communication efficiency and effective features learning through the optimum models sifting.

## 4. Evaluation

In this section, we are inspired by language modeling tasks and conduct a series of experiments to evaluate our FedMed approach. Three baseline methods are implemented, and further experiments are conducted to explore the performance and usability of our proposed FedMed method. The federated learning algorithm is implemented based on PyTorch tools [37].

### 4.1. Datasets

For language modeling, experiments are conducted on three English text datasets to simulate the real-world application of word prediction in federated learning. The datasets are Penn Treebank (PTB) [38,39], Yelp 2013 [40], and WikiText-2 [41]. The three datasets are all appropriate and specific for next-word prediction, and well suited for federated language modeling.

PTB dataset is an extensively annotated text corpus of English from Wall Street Journal material in syntactic level. Larger than PTB, WikiText-2 dataset is a vocabulary collection extracted from verified articles on Wikipedia. Yelp dataset is a detailed dump of business reviews that have a long tail of words each example, so we process and truncate each review within a length of 50 words. Statistics about all three datasets used in the evaluation are shown in Table 1.

### 4.2. Settings

This section depicts the implementation details of our proposed FedMed and settings of groups of experiments we conduct, including comparison with several aggregation methods and communication efficiency evaluation. We introduce three baselines, i.e., FedSGD, FedAvg, and FedAtt, in comparison with our FedMed. Concise definitions upon each baseline and our FedMed are as follows.

- FedSGD: Federated stochastic gradient descent involves all local workers in models aggregation, and local training runs one iteration of gradient descent.
- FedAvg: Global server computes averaging parameters from local workers each round, and local training runs several iterations of gradient descent.
- FedAtt: Global server adopts attentive aggregation for federated optimization each round, and local training keeps a comparable presetting as FedAvg.
- FedMed: Our proposed FedMed develops a novelty aggregation method for federated learning each round, and the local worker keeps a comparable presetting as FedAvg.

To simulate the scene of realistic mobile virtual keyboard prediction, data preprocessing is executed on three public text datasets. Each training dataset is shuffled to randomize the data and avoid the network model’s overfitting. Afterwards, each dataset is divided into 100 (default) subsets, which represent the local data of the maximum number of device workers participating in the federated network for aggregation. According to the specific number of workers, the data subsets are randomly sampled based on the independently identical distribution. The threshold value ϵ is set to be 0.1 for the mediator. Experiments are conducted with federated learning settings by adopting the LSTM-based neural language model, which after reading each word in a sentence, predicts the next word. Our goal is to verify our proposed FedMed compared to FedAvg and FedAtt in terms of federated aggregation and communication efficiency. Therefore, we place little emphasis on better network model as it makes no difference to the FedMed advantage over FedAvg and FedAtt. The hardware backbone uses Nvidia GTX 2080Ti GPU acceleration. The LSTM-based worker model takes a sequence of words as input and generates embeddings into a 300-dimensional word vector space. The embedded words are then fed into two unidirectional LSTM-based model layers, with hidden units of size 400. Lastly, the output of second layer is fed into a fully connected layer to infer next word. SGD is used for optimization and worker models are tuned with decayed learning rate to accelerate model converging.

### 4.3. Results

We take perplexity (PPL) and communication rounds as the evaluation metrics to measure how well the model predicts words and how efficiently the communication behaves. PPL is used widely for word-level language modeling and text generation tasks evaluation. A lower perplexity suggests the model performs better to execute language tasks. PPL is considered to be an intrinsic measure for probabilistic model distribution, which can be given by

(10) $$PPL\left(p\right)≜2^{H(p)}=2^{-\Sigma_{x}\;p\left(x\right)\;\log_{2}\;p\left(x\right)}$$

where H(p) denotes the entropy of the discrete distribution. The lower the PPL, the more accurate the model prediction. Besides, we take an exchange of parameters between server and workers as one communication round. The lower the communication rounds, the more efficient the communication.

#### 4.3.1. Perplexity Comparison

**Figure 4:**
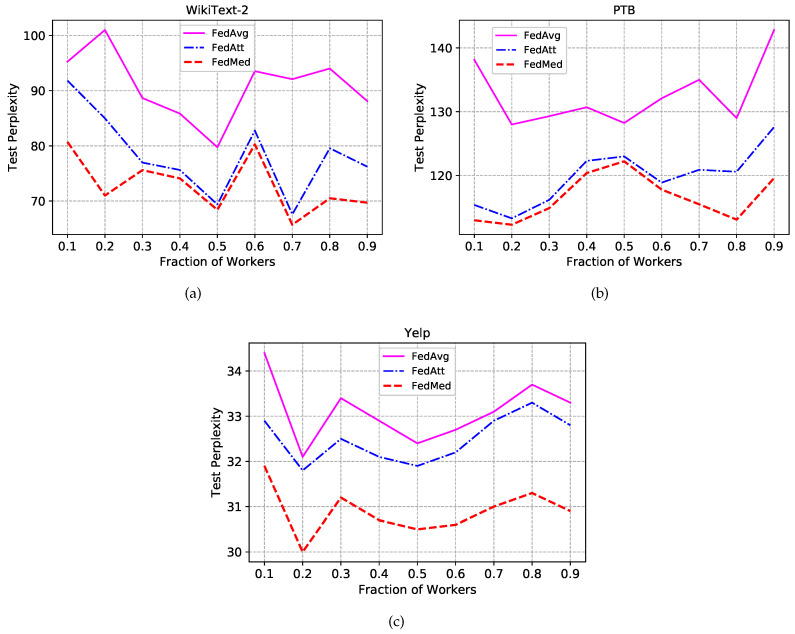
Experimental results of different aggregation methods on test sets. (**a**)Test perplexity on test set of WikiText-2. (**b**)Test perplexity on test set of Penn Treebank. (**c**)Test perplexity on test set of Yelp.

Experiment is first conducted with the 50 round of communication and different worker fraction *C* stands for the size of local workers in parallelism, i.e., C=0.1 indicates there are ten workers training for federated learning. In realistic application scenarios, not all devices are continuously online because of power supply or network breakdown. Hence, it is critical and significant to evaluate the performance of disparate number of workers through altering the fraction *C*. Figure 4 shows the effect of varying *C* for different methods(i.e., FedAvg, FedAtt, FedMed).

The FedMed approach renders a performance boost in all three datasets in all fraction settings, even though it reports a weak advantage over FedAtt when C=0.5 in WikiText-2. Test perplexity does not show discernible trends regularity when fraction *C* varies from 0.1 to 0.9. It also does not report that fewer or more workers can make a contribution to high performance. However, the language model comparatively obtains the best perplexity on the setting of fraction C=0.5. Meanwhile, it can be found that the divergence between the FedMed and the FedAtt is much bigger than the one between the FedAtt and the FedAvg on Yelp dataset, which represents that our FedMed approach achieves more remarkable performance than the two baselines. Concerning the embedding space of Yelp larger than the other two, it can be explained that FedMed provides a considerable generalization while the FedAtt deteriorates a little in terms of generalization on the large dataset. Statistically, we can spot substantial gains: FedMed takes the definite advantage in all three datasets compared to the other two methods.

Furthermore, the model performance is explored on specific fraction settings C=0.1 and C=0.5 respectively. Please note that the fraction of FedSGD equals one theoretically. The results of perplexity for all three datasets are revealed in Table 2. Our FedMed method outperforms the other three methods(i.e., FedSGD, FedAvg, and FedAtt) concerning PPL among all three datasets. When the fraction C=0.5, our proposed approach obtains a better test perplexity compared with fraction C=0.1, which indicates more device workers can culminate in a better PPL to a certain extent. It probably can be explained by the learning process in which more workers join in the federated network and contribute more data on word vector information space. Hence this can avoid models underfitting and improve the performance of global server aggregation.

#### 4.3.2. Communication Efficiency

**Figure 5:**
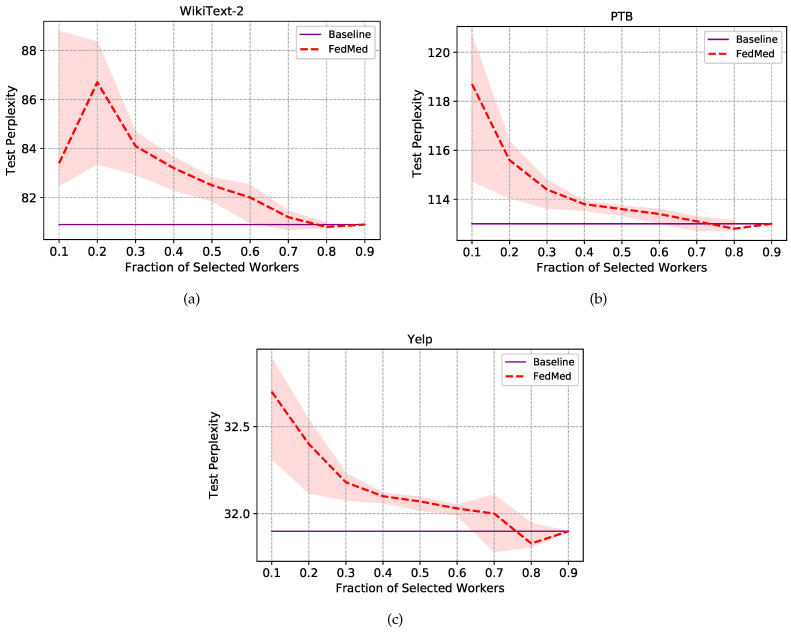
Experimental results of communication efficiency with topK strategy on test sets. The purple line stands for the baseline without topK strategy in three datasets. (**a**)Test perplexity on test set of WikiText-2. (**b**)Test perplexity on test set of Penn Treebank. (**c**)Test perplexity on test set of Yelp.

Besides the comprehensive perplexity, we are also intrigued by the trade-off between communication costs and scales of data storage. The server authorizes a small scale of workers picked from the sets to transfer model parameters for cost reduction. The FedMed is annealed by setting the term fraction β from 0.1 to 0.9. Figure 5 shows the impact of topK strategy on model performance in our proposed FedMed.

For the model evaluation simplification, experiments are conducted under the setting of fraction C=0.1, and other settings are similar to the prior, and topK strategy can get comparable results under different fraction *C* conditions. As is shown in Figure 5, our proposed method obtains considerable improvements in efficiency with less communication cost among all three datasets. To be specific, results on all three datasets can be found that the PPL has a trend of decreasing gradually, which denotes that the server model has better performance, along with more workers selected basically. We can find that the model with topK strategy gets the lowest perplexity on β=0.8, which represents 80% of high-level worker parameters are sifted to upload to the server, and 20% of ones are discarded. What can be seen is that it does not weaken the server model to learn features and predict words. Not only does the method save 20% of communication costs but it gives a little boost for the server’s performance. Using our method, we can conclude that the mediator of FedMed selects high-quality worker models and drops inferior worker models. Hence, the server can get rid of the noise of abnormal data and boost the model’s robustness. Furthermore, when fraction β is 0.5, the test PPL is closer to the purple guideline. That is to say, results are only 0.53%, 1.93%, and 0.54% higher than baselines in WikiText-2, PTB, and Yelp datasets respectively. Nevertheless, the FedMed with topK strategy saves 50% of communication costs, which demonstrates significant boost to the communication efficiency in federated learning.

**Figure 6:**
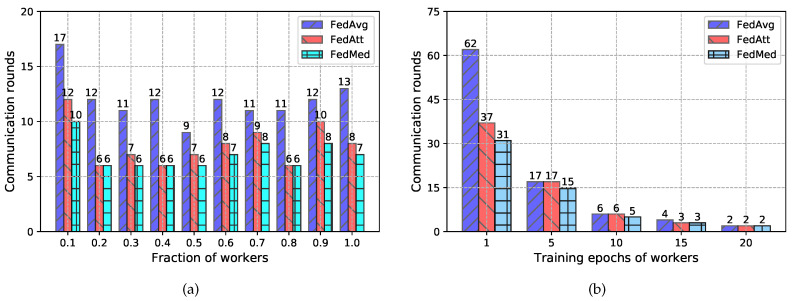
Experimental results of communication rounds with three different methods on WikiText-2 dataset on two factors. (**a**) Communication rounds consumed to achieve the preset performance level through different fractions of workers. (**b**) Communication rounds consumed to achieve the preset performance level through different epochs of workers during training.

Furthermore, the communication round is explored as one communication efficiency metric to simulate and evaluate the bandwidth consumption during model training. Experiments are conducted on the WikiText-2 dataset in terms of two impact factors (i.e., the worker fraction and the epochs of local worker training). Please note that the WikiText-2 dataset is designated as a typical participant due to its pronounced generality rather than anonymous specificity. In the experiments, other conditions are ensured to be the same other than two factors. Experimental results are depicted in Figure 6 where LSTM language model is adopted the same as the prior evaluation does. The test perplexity of threshold of federated training is fixed on 90, which indicates that the processing of federated learning is terminated when PPL is lower than that value. At the moment, the present training rounds is recorded as the communication rounds. According to the Figure 6a, the communication round slightly fluctuates when more and more workers participate in the federated network. However, our FedMed approach performs better than both FedAtt and FedAvg with less communication under most conditions, at least the same with FedAtt when the worker fraction *C* is 0.2, 0.4 and 0.8. FedMed achieves an average of 41.6% and 11.3% lower communication costs compared to FedAvg and FedAtt, respectively. Specifically, FedMed reduces the communication over FedAtt by 20% when the worker fraction *C* is 0.9. In Figure 6b, we also report the communication rounds with respect to the epochs of workers during local computation. To assess the reputation behaviours for all methods with ease, we set the epoch ξ value varied from 1 to 20 in increments of 5 to simulate the effect of different computation preferences on communication. It can be observed that FedMed outperforms all other methods not strictly, achieving an average of 38.4% and 13.8% cost reduction compared to FedAvg and FedAtt, respectively. This denotes that the improvements in efficient communication achieved by FedMed against the other two are significant. In conclusion, topK strategy is first introduced into federated learning for exploiting the communication efficiency. A range of experiments disclose that it is effective against communication reduction and accelerating the model convergence. The advantage of FedMed approach against FedAvg and FedAtt is pronounced both in vertical and horizontal comparison.

## 5. Conclusions

In this paper, we propose a novel FedMed framework to resolve optimization issues in federated learning for mobile keyword prediction. Federated learning discloses a robust heuristic approach and addresses the gap between deep learning and data accessing for artificial intelligence applications, which contributes much towards the engineering field. Nevertheless, for language model neural networks, there are still unsolved issues, especially model aggregation and communication costs to be solved. This FedMed allows us to use adaptive aggregation for zooming in high-quality models, mediation incentive scheme for personalized model collaboration, and topK strategy for progressing communication efficiency. Experimental results among three datasets demonstrate that our proposed FedMed approach provides better performance and considerable improvements.

## Figures and Tables

**Table 1:** Statistical information of datasets used in our experiments. Number stands for the total token size in training, validation and testing of three datasets.

Dataset	Train	Valid.	Test
PTB	887,521	70,390	78,669
Yelp	3,063,578	380,877	424,879
WikiText-2	2,088,628	217,646	245,569

**Table 2:** Test perplexity using LSTM-base model by FedSGD, FedAvg, FedAtt and FedMed methods. The symbol † represents the results disclosed by earlier publications.

Frac.	Method	WikiText-2	PTB	Yelp
1	FedSGD	$112.45{\;}^{†}$	$155.27{\;}^{†}$	38.41
	FedAVG	$95.27{\;}^{†}$	$138.13{\;}^{†}$	34.42
0.1	FedAtt	$91.82{\;}^{†}$	$115.43{\;}^{†}$	32.02
	**FedMed**	**80.32**	**113.03**	**31.90**
	FedAVG	$79.75{\;}^{†}$	$128.24{\;}^{†}$	32.43
0.5	FedAtt	$69.38{\;}^{†}$	$123.00{\;}^{†}$	31.93
	**FedMed**	**68.42**	**122.23**	**30.50**

## Abbreviations

The following abbreviations are used in this paper:

LSTM	Long Short-Term Memory
PPL	Perplexity
JS	Jensen-Shannon
FL	Federated Learning
SGD	Stochastic Gradient Descent
